# Conceptualisation and measurement of child hunger: a rapid review

**DOI:** 10.1017/S1368980026102195

**Published:** 2026-03-25

**Authors:** Alix Mooney, Frédérique Vallières, Greg Sheaf, Maurice Sadlier, Sheila Garry, Andrea Galante, Kristin Hadfield, Azza Warraitch

**Affiliations:** 1 Trinity Centre for Global Health, School of Psychology, Trinity College Dublin, Dublin 2, Ireland; 2 The Library of Trinity College Dublin, Dublin 2, Ireland; 3 World Vision Ireland, The Mews, Garland House, Rathmines Park, Dublin 6, Ireland; 4 World Vision International, Executive Office, Romero House, 55 Westminster Bridge Road, London SE1 7JB, UK

**Keywords:** Child hunger, Child nutrition, Measurement, Conceptualisation

## Abstract

**Objective:**

Child hunger is a significant global health concern prioritised by multiple global public health organisations. In 2006, the US Committee on National Statistics (CNSTAT) highlighted the need for clarity and consistency in the operationalisation and measurement of child hunger. This review examines whether these recommendations have been implemented in child nutrition programming over the past two decades. In addition, we explore how child hunger is currently conceptualised and measured across different contexts.

**Design:**

We conducted a pre-registered rapid review of studies that define or measure ‘child hunger’. Six electronic databases (Web of Science, MEDLINE, Embase, PsycINFO, Social Science Database and ERIC) and websites of twenty public health organisations were searched for reports that mentioned the term ‘child hunger’ or ‘child’ near ‘hunger’ published after 2006.

**Setting:**

There were no restrictions on study settings.

**Participants:**

Studies focusing on children under the age of 18 years were included.

**Results:**

Sixty-seven articles measured child hunger and were therefore eligible for inclusion. Of these, only twenty-three provided a definition of child hunger. Definitions commonly described child hunger as a consequence of or as a subcategory of household ‘food insecurity’. Most scales used in the included studies examined the quantity or amount of food intake by children, while few measures also assessed the quality of food consumed. The physiological dimension of hunger was not measured by any of the questionnaires.

**Conclusions:**

The findings underscore the need for more comprehensive and standardised approaches that account for the multidimensional nature of child hunger.

Defined as ‘a potential consequence of food insecurity that, because of prolonged, involuntary lack of food, results in discomfort, illness, weakness, or pain that goes beyond the usual uneasy sensation’^(1,2)^, hunger affected an estimated 733 million people globally in 2023^(1)^. Among those affected, children are especially vulnerable to hunger^(3)^, with approximately 45 % of all deaths among children under 5 years of age linked to hunger-related concerns^(4)^. Adequate nutrition, particularly in the first 1000 d of life, is thus widely recognised as critical for children’s physical, cognitive and psychological development, as well as long-term health^(4–7)^. Children experiencing hunger are at heightened risk of poor health outcomes, including more frequent illnesses and infections, increased rates of hospitalisation^(7–11)^ and higher odds of asthma and other chronic conditions^(11)^. Moreover, childhood hunger is associated with an increased risk of mental health difficulties during adolescence, including depression and suicidal ideation^(12)^. Beyond individual health consequences, child hunger and undernutrition impose significant economic burdens at the societal level. For example, the estimated annual economic cost of child undernutrition is approximately $600 million in Malawi and $4·7 billion in Ethiopia^(13)^.

Given the profound health and economic consequences of hunger, global efforts have been directed towards its eradication by 2030, as reflected in Sustainable Development Goal (SDG) 2^(14)^. Today, the prevalence of hunger among children varies widely across countries, from as low as 3·0 % in Australia^(6)^ to as high as 58·4 % in Malawi^(7)^. Global crises such as armed conflicts, climate change, economic instability and health pandemics, however, are known to exacerbate food insecurity and its impact on child hunger and malnutrition^(15,16)^. For example, the current humanitarian crisis in Gaza has underscored the devastating effects of conflict on child nutrition, as nearly 75 % of children in food-insecure households reportedly went to bed hungry in 2024^(17)^. The Central African Republic, which has also experienced prolonged conflict and displacement, recorded the highest Global Hunger Index rate in 2023, with one in twenty children reportedly dying before their fifth birthday and 5 % of children suffering from wasting^(16)^. Similarly, Madagascar, which is severely affected by the impacts of climate change, ranked second on the Global Hunger Index, with a child mortality rate of 6·6 in 2023^(16)^. More recently, cuts to funding for humanitarian assistance are expected to further undermine global responses to food insecurity and contribute to rising rates of child hunger in the coming years^(18)^. Amid escalating global food insecurity, a clear understanding of how child hunger is conceptualised and addressed is critical to guide the design, implementation and scale-up of effective child nutrition interventions. Existing programmes targeting child hunger adopt varying approaches to its conceptualisation and measurement, with some equating child hunger with malnutrition or food insecurity. While these concepts are closely related^(2)^, using them interchangeably can result in misaligned policies and ineffective interventions. Food insecurity, for instance, is a household-level construct which reflects whether a family has consistent access to adequate food and does not necessarily capture the individual experiences of hunger, particularly among children^(2)^. Within food-insecure households, for example, parents may try to shield children from hunger by prioritising their child’s food intake. Child hunger may therefore be a separate and more severe indicator of food insecurity^(19)^. Failing to distinguish between the two, however, obscures the true extent of child hunger and can result in interventions that fail to target the children most in need.

Aware of this gap, a panel convened by the US Committee on National Statistics (CNSTAT) recommended that child hunger and household food insecurity be measured separately, concluding that for valid measurement of hunger, it is essential to first define and operationalise child hunger^(2)^. This clarity in the conceptualisation of child hunger would facilitate more accurate and consistent measurement across contexts and over time, thereby supporting the implementation and evaluation of programmes aimed at reducing child hunger. However, limited research has investigated whether and, if so, how these recommendations have been reflected in child nutrition research over the past two decades. To address this gap, we conducted a review of the literature on ‘child hunger’ to examine how it is most commonly conceptualised and measured. This review was guided by the following research questions: (i) How is child hunger defined and conceptualised? and (ii) which questionnaires are commonly used to measure child hunger?

## Methods

Recommended for the production of timely evidence for decision-making^(20)^, we conducted a pre-registered rapid review in accordance with the Interim Guidance by the Cochrane Rapid Reviews Methods Group^(20)^. Findings are reported in line with the PRISMA guidance for systematic reviews^(21)^, as the PRISMA extension for rapid reviews is currently under development.

### Eligibility criteria

We included peer-reviewed articles and grey literature reports that focused on hunger among children aged 0–18 years. To be included, papers had to mention the terms ‘child hunger’ or ‘child’ and ‘hunger’ together or in close proximity. Articles were excluded if they did not provide either a definition of child hunger or a description of the methods used to measure child hunger in the study. The full inclusion and exclusion criteria are shown in Table 1.

**Table 1.** Inclusion and exclusion criteria

	Inclusion criteria	Exclusion criteria
Population	Studies focusing on children ≤ 18 years.	Studies focusing on both children and adults without providing segregated data for children ≤ 18 years.
Intervention	Programmes, interventions, surveys, observational studies that aim to address, understand or measure ‘child hunger’.	Programmes, interventions or observations that specifically aim to address, understand or measure child malnutrition, undernutrition, overnutrition, micronutrient deficiency, obesity, without explicitly mentioning the term ‘child hunger’.
Main outcome(s)	• Definitions of child hunger. • Measures or methods used to assess child hunger.	–
Other	• Studies published from the year 2006 onwards. • Studies published in English Language.	Conference abstracts, editorials and articles for which full texts are not available after contacting the corresponding author.

### Information sources and search strategy

We searched for peer-reviewed literature published from 2006 onwards across six electronic databases: Web of Science, MEDLINE, Embase, PsycINFO, Social Science Database and ERIC. The search strategy for the review included keywords for the population (children 18 years or younger) and the condition under study (child hunger). The search strategy was developed with the assistance of GS, a subject librarian. The initial search was conducted in January 2024 and subsequently updated in February 2025, with newly identified records imported into the existing Covidence account used for this review. Annex 1 contains an example search strategy formatted for Web of Science.

As per the Cochrane guidelines for rapid reviews, a comprehensive grey literature search is not required for rapid review^(20)^. However, given that child hunger is a key priority area for numerous global and non-profit organisations, a targeted grey literature search was considered essential to identify reports specifically addressing the conceptualisation, evaluation or measurement of ‘child hunger’. Accordingly, a systematic grey literature search was conducted on the websites of the following organisations: WHO, UNICEF, Scaling Up Nutrition, GAIN Health, ReliefWeb, World Food Programme, Save the Children, Akshaya Patra, Feeding America, Action Against Hunger, The Hunger Project, Care, Food for the Hungry, Caritas, Mercy Corps, Inter-agency Network for Education in Emergencies, Global Hunger Index, Global Nutrition Report, Core Group, Emergency Nutrition Network and Advancing Nutrition. These organisations were selected in collaboration with non-profit organisations implementing nutrition programmes aimed at addressing child hunger.

### Study selection

Search results for peer-reviewed literature retrieved from electronic databases were imported into Covidence, while grey literature identified through organisational websites was recorded in an Excel spreadsheet. Title and abstract screening, as well as full-text screening, were conducted by AM for the initial search conducted in 2024 and by AW for the updated search conducted in 2025. To ensure quality control and resolve any discrepancies, all results were reviewed and discussed with a third author (KH).

### Data extraction and synthesis

A data extraction form was developed in Covidence to systematically collect the following information: author(s) and year of publication, country of study, definitions of child hunger, study methodology, measurement tools used to assess child hunger and demographic characteristics of study participants (age range and gender). Relevant data from peer-reviewed literature were extracted in Covidence, while data from grey literature reports were extracted using Excel. AM independently completed all data extraction for studies included from the 2024 search, while AW extracted data from studies identified through the updated search in 2025. A randomly selected subset of five articles was reviewed by KH to promote rigour.

All extracted data were compiled in Excel for analysis. Data pertaining to the definitions of child hunger, measurement methods and studied population groups were analysed using narrative synthesis. Relevant fields were inductively coded and categorised under emerging themes to narratively describe the key patterns within the data.

### Quality assessment

Given that this rapid review was focused on mapping concepts and methodologies related to child hunger rather than evaluating the effectiveness of interventions, a quality assessment of the included studies was not conducted. The decision to include studies regardless of quality allowed for a more comprehensive and inclusive assessment of how research studies define and measure child hunger.

## Results

**Figure 1.**
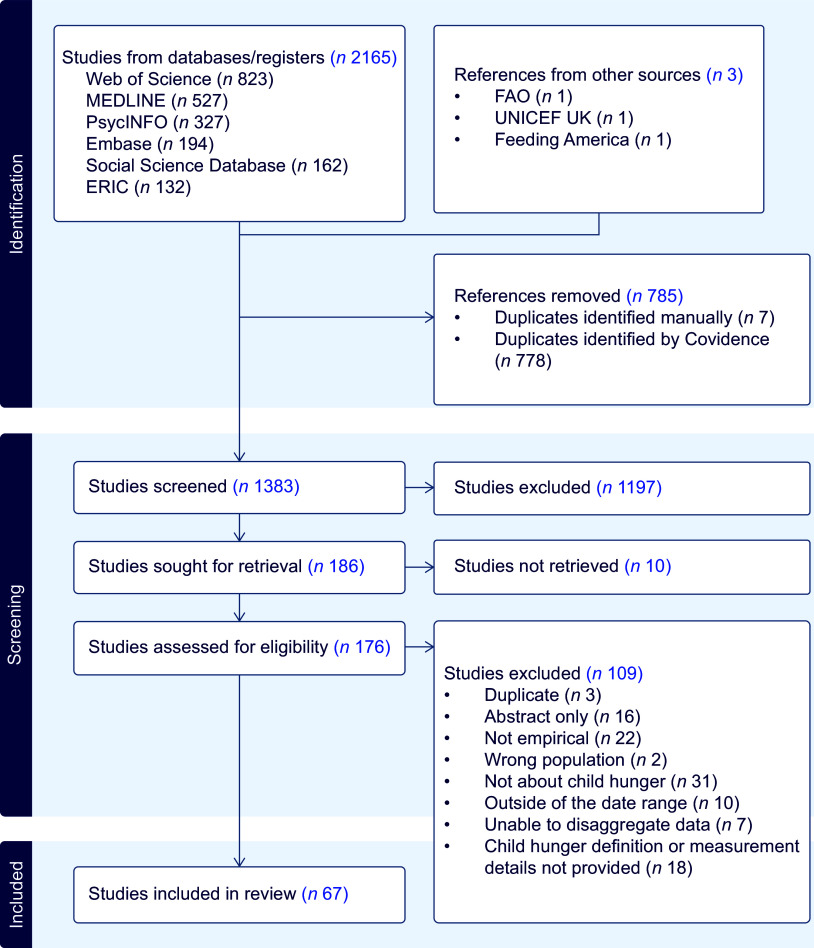
PRISMA flow chart.

We included a total of sixty-seven eligible studies from the initial 2165 reports identified from the electronic databases and other sources. Figure 1 summarises the study selection process.

### Sample characteristics

Most studies were conducted in the USA (*n* 19), followed by Malaysia (*n* 9), Canada (*n* 8) and South Africa (*n* 4) (Table 2).

**Table 2.** Included studies (*n* 67)

Study	Stated age of population	Country	Questions answered by
Alaba *et al.*, 2022^(22)^	0–18 years	South Africa	Parents/caregivers
Apodaca *et al.*, 2010^(23)^	< 5 years	Global	Third party
Azhar *et al.*, 2023^(24)^	< 12 years	USA	Parents/caregivers
Baker *et al.*, 2024^(6)^	3–8 years	Australia	Teachers
Bennett *et al.*, 2014^(25)^	6–9 years	UK	Children
Bernal *et al.*, 2012^(26)^	10–17 years	Venezuela	Children
Canbolat *et al.*, 2023^(27)^	13–14 years (grade 8)	40 countries	Children
Canbolat *et al.*, 2024^(28)^	Grade 8 students	38 countries	Children
Cumiskey *et al.*, 2024^(29)^	11–15 years	Canada	Children
De Amorim *et al.*, 2021^(30)^	11–19 years – (focusing on data 11–15 years)	Brazil	Children
Depboylu & Simsek, 2025^(31)^	10–14 years	Turkey	Children
Dlamini *et al.*, 2024^(32)^	< 17 years	South Africa	Caregivers
Drucker *et al.*, 2019^(33)^	< 18 years	USA	Caregivers
Ecker *et al.*, 2013^(34)^	5–12 years (kindergarten to sixth grade)	USA	Children
Findlay *et al.*, 2013^(35)^	2–5 years	Canada	Parents
Goni *et al.*, 2024^(36)^	< 18 years	Malaysia	Caregivers
Gordon *et al.*, 2012^(37)^	< 18 years	USA	Household head – parents/caregivers
Hadley *et al.*, 2006^(38)^	0–5 years	USA	Parents/caregivers
Hamid *et al.*, 2022^(39)^	0–5 years	Malaysia	Parents (mothers)
Hammami *et al.*, 2020^(40)^	11–16 years (grade 6–10)	Canada	Children
Haque *et al.*, 2017^(41)^	6–59 months	Bangladesh	Parents/caregivers
Harper *et al.*, 2022^(42)^	0–17 years	South Africa	Parents/caregivers
Heflin *et al.*, 2012^(43)^	61 months	USA	Caregivers
Henjum *et al.*, 2019^(44)^	< 18 years	Norway	Parents/caregivers
Hicks *et al.*, 2024^(45)^	< 18 years	USA	Caregivers
Janda-Thomte *et al.*, 2024^(46)^	14–17 years	USA	Children
Jiménez-Cruz *et al.*, 2007^(47)^	6–12 years	Mexico	Children
Jones *et al.*, 2018^(48)^	< 18 years	USA	Parents/caregivers
Kennedy *et al.*, 2013^(49)^	0–17 years	USA	Parents/caregivers
Kersey *et al.*, 2007^(50)^	< 3 years	USA	Caregivers
Kesternich *et al.*, 2015^(51)^	0–3, 4–10 and 0–16 years	Germany	Children
Kirkpatrick *et al.*, 2010^(11)^	10–15 years	Canada	Person most knowledgeable (PMK) about the child
Korinek *et al.*, 2024^(52)^	< 15 years	Vietnam	Children
Krupsky *et al.*, 2022^(53)^	14–18 years (9th–12th grade)	USA	Children
Li *et al.*, 2025^(54)^	< 17 years	China	Children
Liu *et al.*, 2021^(55)^	12–15 years	41 countries	Children
Liu *et al.*, 2022^(56)^	12–15 years	60 countries	Children
McIntyre *et al.*, 2012^(57)^	2–9 years	Canada	Parents/caregivers
McLaren *et al.*, 2018^(58)^	< 2 years	South Africa	Parents/caregivers
Metallinos-Katsaras *et al.*, 2009^(59)^	1 month–5 years	USA	Parents/caregivers
Mwambene *et al.*, 2013^(60)^	15 years and under	Malawi	Children
Nalty *et al.*, 2013^(61)^	6–11 years	USA	Parents (mothers)
Naser *et al.*, 2014^(62)^	2–12 years	Malaysia	Parents (mothers)
Ooi *et al.*, 2023^(7)^	6 months–7 years	Malaysia	Parents/caregivers
Pilkay *et al.*, 2024^(63)^	0–1, 3, 5, 9, 15 years	USA	Mothers
Rahman *et al.*, 2021^(64)^	< 5 years	Malaysia	Parents
Rengarajoo *et al.*, 2023^(65)^	2–11 years	Malaysia	Parents
Romo *et al.*, 2016^(66)^	13–17 years (grades 8–11)	Ecuador	Children
Sambu *et al.*, 2019^(67)^	0–17 years	Africa	Adult
Seager *et al.*, 2023^(68)^	10–18 years	Bangladesh	Children
Sebileau *et al.*, 2025^(69)^	Not reported	New Zealand	Not reported
Sentenac *et al.*, 2016^(70)^	11–16 years (grades 6–10)	Canada	Children
Sharkey *et al.*, 2013^(71)^	< 18 years	USA	Parents (mothers)
Sheikh *et al.*, 2024^(72)^	10–19 years	Pakistan	Children
Shoae *et al.*, 2007^(73)^	1–18 years	Iran	Parents (mothers)
Smith *et al.*, 2008^(74)^	9–18 years	USA	Children
Smith *et al.*, 2023^(75)^	12–15 years	Africa, Asia and America	Children
Steyer *et al.*, 2023^(19)^	5–6 years	USA	Teachers
Stevenson *et al.*, 2024^(76)^	1–12 years	Australia, Canada, UK, New Zealand, Sri Lanka, Ireland, South Korea and USA	Caregivers
Swahn *et al.*, 2012^(77)^	13–16 years	Botswana, Kenya, Uganda and Zambia	Children
Tam *et al.*, 2014^(78)^	0–5 years	Canada	Parents/caregivers
Tay *et al.*, 2023^(79)^	13–18 years	Malaysia	Children
Teh *et al.*, 2020^(80)^	7–11 years	Malaysia	Children
Vale *et al.*, 2022^(81)^	10–14 years	Brazil	Children
Weffort *et al.*, 2024^(82)^	0–18 years	Global	Multiple raters
Wong *et al.*, 2014^(83)^	0–5 years	Malaysia	Parents (mothers)
Zhang *et al.*, 2024^(84)^	< 18 years	China	Children

### Conceptualisation of child hunger

Out of the sixty-seven studies included in the review, twenty-three provided a specific definition of child hunger (see Table 3). Several key themes emerged from these definitions.

**Table 3.** Child hunger definitions extracted from included articles (*n* 23)

Studies	Child hunger definitions
Apodaca *et al.*, 2010^(22)^	Child hunger is the result of children’s lack of access to food rather than a deficiency of available food. Children go hungry in countries and households where food is plentiful.
Azhar *et al.*, 2023^(23)^	A subcategory within food insecurity is childhood hunger, which refers to an inadequate amount of food intake of children, due to insufficient economic, family or community resources.
Ecker *et al.*, 2013^(24)^ and Hicks *et al.*, 2024^(25)^	Hunger is an individual-level physiological condition that is potentially caused by food insecurity (USDA Definitions of Food Security 2022).
Depboylu & Simsek, 2025^(41)^	Homeostatic hunger is a biological response to acute energy depletion, signifying an actual or impending energy deficit. Hedonic hunger is defined as the urge to eat and enjoy food even when there is no physiological hunger.
Haque *et al.*, 2017^(26)^	Situations, when someone cannot acquire adequate amount of food even for a short duration is described as hunger.
Jones *et al.*, 2018^(27)^	The US Department of Agriculture (USDA) defines very low food security in children, or child hunger, as having experienced at least five of the following factors: (1) diminished quality and size of meals, (2) limited variety of foods, (3) skipped meals, (4) not eaten for a whole day, (5) gone hungry because their caregiver did not have enough money to buy food or (6) some of these conditions for more than a 3-month period.
Kersey *et al.*, 2007^(28)^ and Liu *et al.*, 2022^(29)^	Hunger is defined by the USDA as the uneasy or painful sensation caused by a lack of food, the recurrent and involuntary lack of access to food.
Kirkpatrick *et al.*, 2010^(11)^	Hunger is the most extreme manifestation of food insecurity.
Li *et al.*, 2025^(40)^	Hunger also comprises a lack of essential vitamins and minerals and the negative effects of this hidden hunger on health.
McIntyre *et al.*, 2012^(30)^	Child hunger is an extreme manifestation of food insecurity and is linked to inadequate dietary intake, putting these children at risk for poor health.
Naser *et al.*, 2014^(31)^	Child hunger (or severe food insecurity) is the most severe problem arising from household food insufficiency and is characterised by decreases in the quantity of food eaten by children.
Ooi *et al.*, 2023^(7)^	Hunger is defined as an uneasy or painful sensation caused by a lack of food. Its measurement captures the severity of deprivation of a basic need from resource constraints. Thus, child hunger implies an extensive reduction in food intake for all household members to the extent that children also repeatedly experience hunger.
Romo *et al.*, 2016^(32)^	Hunger occurs at the individual level and is a physiological condition that can result from severe food insecurity. Hunger in this study was defined as having gone hungry in the past 30 d due to lack of food in the home.
Sharkey *et al.*, 2013^(33)^	The Community Childhood Hunger Identification Project (CCHIP) defined hunger as the mental and physical condition that comes from not eating enough food due to insufficient economic, family or community resources.
Smith *et al.*, 2008^(34)^	Food insecurity may lead to, or be associated with, hunger, a prolonged involuntary lack of food that results in discomfort, illness, weakness or pain that goes beyond the usual uneasy sensation.
Smith *et al.*, 2023^(35)^	Hunger is an uncomfortable or painful physical sensation that can be due to severe food insecurity.
Stevenson *et al.*, 2024^(42)^	Many people can determine if they are hungry or thirsty by reference to their internal state. Hunger, an empty rumbling or growling stomach, and irritability or fatigue are often reported as signs indicating that food will be rewarding to eat now.
Tam *et al.*, 2014^(36)^	Hunger is an extreme form of food insecurity, which may also be referred to as very low food security.
Weffort *et al.*, 2024^(37)^	Josué de Castro defined as total hunger, the true famishment that English-speaking people call starvation, a phenomenon, in general, limited to areas of extreme poverty and exceptional contingencies, as the much more frequent and more serious phenomenon; and the partial form, the so-called hidden hunger, in which, due to the permanent lack of certain nutritional elements, in a habitual diet, entire population groups allow themselves to slowly die of hunger, despite eating every day. According to the WHO, hidden hunger is defined as the non-explicit need for one or more of the twenty-six micronutrients that are essential for adequate body function.
Wong *et al.*, 2014^(38)^	Child hunger indicates the most severe form of food insecurity since adult caregivers are assumed to cut down their own food intake first in order to maintain an adequate food provision for their children.
Zhang *et al.*, 2024^(39)^	The childhood hunger experiences (CHE) refers to the situation of facing shortages of food and supplies during childhood and being exposed to malnutrition for a long time.

The text included in the column to the right is directly quoted from the included article.

#### Centrality of food insecurity, quantity and quality

A total of twenty studies defined child hunger as being associated with a lack of access to food or food insecurity^(7,11,22–39)^. Among these, fifteen studies conceptualised child hunger as an outcome of food insecurity^(7,11,22–25,28–35,37)^, while five studies equated child hunger directly with food insecurity^(26,27,36,38,39)^. This pattern reflects a conceptual model whereby child hunger is both a symptom and an extreme manifestation of inadequate food access.

In addition, eleven studies defined child hunger as inadequate food consumption. Nine of these studies consistently identified reduced or insufficient food intake, often reflected in smaller meal portions or skipped meals, as a core indicator of childhood hunger^(23,26,27,30,31,33,37,40,41)^. A subset of these studies also emphasised the role of decreased food quality as a contributing factor to childhood hunger^(27,41)^. In these studies, low food quality was characterised in two ways: first, as a lack of dietary variety^(27)^, and second, as the consumption of foods deficient in essential nutrients and vitamins, resulting in hidden hunger^(37,41)^.

#### Physiological dimensions of child hunger

The underlying physical and psychological aspects of child hunger were highlighted in definitions used in ten studies^(7,24,25,28,29,32–35,42)^. Amongst these ten studies, the predominant focus was on physiological framing of hunger^(24,25,32)^ evidenced by definitions describing hunger as an ‘uneasy or painful sensation’^(7,28,29,35)^, or a state of ‘discomfort, illness, and weakness, or pain that goes beyond the usual uneasy sensation’^(34)^. Only one study incorporated the psychological dimensions in the definition of childhood hunger, particularly in relation to mental health consequences stemming from chronic hunger^(33)^.

#### Operationalisation of duration or frequency of hunger

The temporal dimension of hunger was inconsistently addressed across the reviewed definitions. Some studies provided specific time frames, such as ‘more than three months’ or ‘within the past month’^(27,32)^, which may imply a focus on the chronic nature of hunger. In contrast, other definitions used time frames that referred to more episodic experiences of hunger, describing situations in which children were unable to access food even for ‘short periods’^(26)^. A few studies used vague descriptors such as ‘recurrent or repeated’^(7,28,29)^ or ‘long-term’ experiences of hunger^(39)^ without clarifying the precise duration, frequency or severity of hunger episodes.

#### Individual-level conceptualisation of child hunger

Only three studies explicitly defined childhood hunger as an individual-level construct that was distinct from the household-level construct of food insecurity^(24,25,32)^.

### Measurement of child hunger

In the included studies, child hunger was assessed using a total of twenty-one measures. These measures are outlined in Table 4.

**Table 4.** Measures of child hunger

Tool used to measure child hunger	Assesses quality of food intake	Assesses frequency of hunger	Indicators measured for child hunger	Measurement level	Time frame	Rater(s)	Authors
Radimer/Cornell Hunger and Food Insecurity Instrument^(43)^	Yes	Yes	♣ Low quality of food due to limited resources ♣ Inadequate food consumption due to limited resources ♣ Experiencing hunger due to limited resources	Individual and household	None	Caregiver-reported	Goni *et al.*, 2024^(77)^ Hadley *et al.*, 2006^(78)^ Hamid *et al.*, 2022^(79)^ Henjum *et al.*, 2019^(80)^ Naser *et al.*, 2014^(31)^ Ooi *et al.*, 2023^(7)^ Rengarajoo *et al.*, 2023^(81)^ Sharkey *et al.*, 2013^(33)^ Shoae *et al.*, 2007^(82)^ Tay *et al.*, 2023^(83)^ Teh *et al.*, 2020^(84)^ Wong *et al.*, 2014^(38)^
Question – ‘During the past 30 days, how often did you go hungry because there was not enough food in your home?’ Modified question for hunger during Covid-19 – during the COVID-19 pandemic, how often did you go hungry because there was not enough food in your home?	No	Yes	♣ Frequency of hunger due to food insecurity	Individual	30 d	Child-reported	Alaba *et al.*, 2022^(49)^ Amorim *et al.*, 2021^(50)^ Janda-Thomte *et al.*, 2024^(51)^ Krupsky *et al.*, 2024^(52)^ Liu et al, 2022^(53)^ Liu *et al.*, 2022^(29)^ Mwambene *et al.*, 2013^(54)^ Romo *et al.*, 2016^(32)^ Smith *et al.*, 2023^(35)^ Swahn *et al.*, 2012^(55)^ Vale *et al.*, 2022^(56)^
US Household Food Security Survey Module (HFSSM)^(46)^/modified or adapted version of the US HFSSM.	Yes	Yes	♣ Low quality of food due to limited resources ♣ Reduced food intake due to limited resource ♣ Inadequate food consumption due to limited resources ♣ Experiencing hunger due to limited resources	Individual and household	12 months	Caregiver-reported	Drucker *et al.*, 2019^(85)^ Gordon *et al.*, 2012^(86)^ Jones *et al.*, 2018^(27)^ Kennedy *et al.*, 2013^(87)^ Kersey *et al.*, 2007^(28)^ Metallinos-Katsaras *et al.*, 2009^(88)^ Nalty *et al.*, 2013^(89)^ Smith *et al.*, 2008^(34)^
Community Childhood Hunger Identification Project (CCHIP) index^(47)^	No	Yes	♣ Lack of access to food due to limited resources ♣ Inadequate food consumption due to limited resources ♣ Experiencing hunger due to limited resources ♣ Sleeping hungry due to limited resources	Household	12 months	Caregiver-reported/child-reported	Azhar *et al.*, 2024^(23)^ Dlamini *et al.*, 2024^(74)^ Jiménez-Cruz *et al.*, 2007^(75)^ McLaren *et al.*, 2018^(76)^
Question – ‘Has this child ever experienced being hungry because the family has run out of food or money to buy food?’ One study used a variation of this question: How often did you not eat because there was not enough money to buy food? Two studies also included two follow-up questions to collect additional information. If yes, how often? How do you cope with feeding your child when this happens?	No	Yes	♣ Experiencing hunger due to food insecurity/frequency of hunger	Individual	30 d/ever	Caregiver-reported (*n* 5)/child-reported (*n* 1)	Kirkpatrick *et al.*, 2010^(11)^ Tam *et al.*, 2014^(36)^ Sebileau *et al.*, 2025^(58)^ Findlay *et al.*, 2013^(59)^ McIntyre *et al.*, 2012^(30)^
Question about overall experience of hunger (Looking back at your life), was there a period when you suffered from hunger?/In the past year did an adult/child go hungry?/how frequently children in the household went hungry over a 30-d period/Did you experience severe hunger (e.g. going to bed hungry, having to scavenge for food) for a month or longer due to food shortage during your childhood.	No	Yes	♣ Duration/frequency of hunger	Individual/Household	30 d/12 months/open-ended	Child-reported (*n* 2)/caregiver-reported (*n* 2)	Kesternich *et al.*, 2015^(60)^ Harper *et al.*, 2022^(69)^ Sambu *et al.*, 2019^(70)^ Korinek *et al.*, 2024^(61)^
Other^*^	No	Yes	♣ Frequency of hunger and food insecurity/stunting/inadequate food consumption due to limited resources/responsiveness to hunger	Household/individual/individual/individual	12 months/not reported/not reported/in the past few weeks	Caregiver-reported	Rahman *et al.*, 2021^(71)^ Apodaca., 2010^(22)^ Pilkay *et al.*, 2024^(62)^ Stevenson *et al.*, 2024^(42)^
Question – ‘Some young people go to school or to bed hungry because there is not enough food at home. How often does this happen to you?’	No	Yes	♣ Frequency of hunger due to food insecurity	Individual	None	Child-reported	Cumiskey *et al.*, 2024^(63)^ Hammami *et al.*, 2020^(64)^ Sentenac *et al.*, 2016^(65)^
Question – How often (every day, almost every day, sometimes, never) you feel hungry when you arrive at school.	No	Yes	♣ Frequency of hunger	Individual	None	Child-reported	Baker *et al.*, 2024^(6)^ Canbolat *et al.*, 2023^(66)^ Canbolat *et al.*, 2024^(67)^
Question – When you were a child before age 17 was there ever a time when your family did not have enough food to eat?	No	No	♣ Food insecurity or lack of adequate food, inadequate food consumption	Household	Before the age of 17	Child-reported	Li *et al.*, 2025^(40)^ Zhang *et al.*, 2024^(39)^
Picture measures	Yes	No	♣ Severity of hunger ♣ Food security (having access to enough food) ♣ Severity of hunger	Individual and household/individual	Weekend/not reported	Child-reported	Ecker *et al.*, 2013^(24)^ Bennett *et al.*, 2014^(57)^
Household Food Insecurity Access Scale (HFIAS)^(48)^	Yes	No	♣ Worrying about food insecurity ♣ Low quality of food due to restricted diet ♣ Limited and inadequate food intake ♣ Experiencing hunger	Individual and household	4 weeks	Caregiver-reported	Haque *et al.*, 2017^(26)^ Sheikh *et al.*, 2024^(90)^
Food security battery of questions	No	Yes	♣ Frequency of reduced food intake ♣ Inadequate food intake	Household	12 months	Caregiver-reported	Canbolat *et al.*, 2023^(66)^ Arteaga *et al.*, 2012^(72)^
Household Hunger Scale (HHS)^(25)^	No	No	♣ Severity of hunger ranging from little to severe	Household	Not reported	An adult member of the household	Hicks *et al.*, 2024^(25)^
Modified/adapted version of Radimer/Cornell Hunger and Food Insecurity Instrument for an interview^(73)^	No	No	♣ Food security ♣ Situations and factors contributing to food insecurity ♣ Previous experiences of hunger ♣ Coping strategies for hunger	Household	Not reported	Child-reported	Bernal *et al.*, 2012^(73)^
Kindergarten Readiness Inventory (KRI)	No	Yes	♣ Frequency of hunger	Individual	Not reported	Teacher-reported	Steyer *et al.*, 2023^(19)^
Power of Food Scale	No	No	♣ Hedonic Hunger	Individual	Not reported	Child-reported	Depboylu & Simsek, 2025^(41)^
Food and Nutrition Technical Assistance (FANTA) Project’s Food Insecurity Access Scale (FIAS)	Yes	Yes	♣ Frequency of hunger due to limited resources ♣ Amount of food intake ♣ Low quality of food consumed	Individual	4 weeks	Child-reported	Seager *et al.*, 2023^(68)^

*Rahman *et al.*, 2021 – Structured questionnaire relating to household dietary diversity and household food security was used to collect data from the respondents. The questionnaire was adopted and adapted from various questionnaires based on the study’s objectives. Apodaca *et al.*, 2010 – The prevalence of stunted children under the age of five is used to measure child hunger and malnutrition. Pilkay *et al.* (2024) asked mothers about the meals missed by children. Stevenson *et al.* (2024) administered a 25-item measure on caregivers’ attention towards, and response to, their child’s interoceptive hunger and thirst cues.

#### Overview of the commonly used measures for child hunger

The most frequently used measure in the included studies (*n* 12) was the Radimer/Cornell Hunger and Food Insecurity Instrument^(43)^. This instrument includes ten items, with three questions specifically focusing on child hunger: Question 8 relates to the quality of a child’s diet, and questions 9 and 10 focus on the quantity of food intake. Child hunger was assumed if questions 9 and 10 were answered with either ‘sometimes true’ or ‘often true’^(44,45)^. Other frequently used and validated survey tools included the US Household Food Security Survey Module (HFSSM)^(46)^ (*n* 8), the Community Childhood Hunger Identification Project (CCHIP) index^(47)^ (*n* 4) and the Household Food Insecurity Access Scale (HFIAS)^(48)^ (*n* 2). The HFSSM is an eighteen-item questionnaire that measures food security, with ten questions focusing on the quality and quantity of food intake as indicators of child hunger^(46)^. The CCHIP is an eight-item measure that assesses food insecurity and hunger among low-income families with at least one child under the age of 12 years^(47)^. Four of the CCHIP items are aimed to capture various indicators of child hunger resulting from food insecurity. The HFIAS is a nine-item tool that assesses both food insecurity and the resulting child hunger manifested in the form of reduced food intake, consumption of low-quality food restricted in variety and disrupted eating patterns^(48)^.

Twenty-eight studies assessed child hunger using a single-item measure. The most commonly used one-item measure was: ‘During the past 30 days, how often did you go hungry because there was not enough food in your home?’ (*n* 11)^(29,32,35,49–56)^. Possible responses were ‘never’, ‘rarely’, ‘sometimes’, ‘most of the time’ and ‘always’. Two surveys used words and pictures to measure child hunger among school students. Bennett *et al.* (2014)^(57)^. developed a picture rating scale that consisted of five cartoon bears with different amounts of food in their stomachs, represented by black ovals. The ovals would increase in size in proportion to the amount of food consumed and the bear’s level of satisfaction. Each of the cartoon bears were accompanied by a label to describe the bear’s level of hunger, starting from ‘very hungry’ to ‘not hungry at all’^(57)^. Similarly, Ecker *et al.* (2013)^(24)^ used a hunger survey that contained words and pictures to make it easier for all participants to understand the survey as some students were English Language Learner students or were unable to read. This study used labelled cartoon faces to depict hunger and satiety from 1 ‘not hungry’, 2 ‘a little hungry’ to 3 ‘very hungry’^(24)^.

#### Measurement levels and time frames

A total of twelve scales were used across thirty-one studies^(6,11,19,22,29,30,32,35,36,41,42,49–68)^ to measure child hunger at the *individual child level*. Of these, seven scales used in twenty-two studies were self-rated by children^(6,29,32,35,41,49–57,60,61,63–68)^, one questionnaire in a single study was rated by teachers^(19)^ and three measures used in three studies were completed by caregivers^(22,42,62)^. One measure was completed by children in one study^(58)^ and by caregivers in four studies^(11,30,36,59)^. At the *household level*, seven scales were used to assess food insecurity and child hunger in thirteen studies^(23,25,39,40,66,69–76)^. Among these, four questionnaires were rated by caregivers in six studies^(25,66,69–72)^, two measures were child-rated in three studies^(39,40,73)^, while one tool was completed by either children or caregivers in four studies^(23,74–76)^.

Twenty-three studies measured food insecurity and child hunger at both *the individual and household level*s simultaneously^(7,24,26–28,31,33,34,38,77–90)^, using four different questionnaires. Three of these scales were rated by caregivers in twenty-two studies^(7,26–28,31,33,34,38,77–90)^, while one scale was child-rated in a single study^(24)^. The time frames used to measure child hunger varied widely across studies. The most frequently used reference periods were the past 12 months (*n* 15)^(23,27,28,34,69,71,72,74–76,85–89)^ and the past 30 d (*n* 15)^(26,29,32,35,50,52–56,58,61,68,70,90)^, though other recall periods were also used in studies.

#### Constructs measured by the child hunger measures

Five key constructs were captured by the scales used in the included studies: food insecurity and hunger (*n* 11), hunger (*n* 7), food insecurity (*n* 2), hedonic hunger (*n* 1) and stunting (*n* 1) (Table 4). These five constructs were measured using nine distinct indicators (online Supplementary Table 1). Most of these scales (*n* 14) claimed to assess the overall experience of hunger among children without defining or operationalising hunger. In nine of these scales, hunger was defined as a consequence of food insecurity or resulting from a lack of resources to acquire food.

## Discussion

This rapid review aimed to explore how ‘child hunger’ is currently conceptualised and measured, identifying considerable methodological variation and inconsistencies in the definitions and measurement of child hunger across included studies. First, most studies defined and measured child hunger as a *consequence* of food insecurity. This is aligned with the results from a previous review examining various measures of food insecurity, where most questionnaires were found to include items on child hunger as an indicator of food insecurity^(91)^. A few studies used ‘child hunger’ and ‘food insecurity’ *interchangeably* in their definitions of child hunger^(26,27,36,38,39)^. This conflation is highly contested in the literature, as some studies argue that ‘hunger’ manifests only in cases of very low food security^(92)^, while others, such as Steyer *et al.* ^(19)^, contend that children may be shielded from hunger by adults even in households experiencing high food insecurity. The latter perspective aligns with the recommendations of the CNSTAT, which advocated for the distinct conceptualisation and measurement of hunger and food insecurity^(2)^. Consistent with this guidance, most scales either assessed hunger (*n* 7) or food insecurity (*n* 2) or measured both constructs as related yet distinct concepts (*n* 11).

CNSTAT further recommended the need to operationalise hunger to enable consistent and valid measurement^(2)^. Among the studies that provided an operationalised definition of hunger, most (*n* 9) focused on quantitative indicators such as number of meals skipped, portion size reductions or the number of days a child lacked adequate food, while the quality of food consumed was rarely discussed^(27,41)^. This pattern is similarly reflected in the measurement of child hunger, with most scales assessing the amount of food intake. This disproportionate emphasis on quantity-based indicators of child hunger over the quality of food consumed is consistent with a conceptualisation of hunger that fails to take into account ‘hidden hunger’; adequate access and intake of nutrient-poor foods may shield children from overtly experiencing hunger, yet leaves them vulnerable to stunting, cognitive delays and a weakened immune system^(37)^. Consequently, micronutrient deficiencies characteristic of hidden hunger are not captured in measurement tools solely focused on food access or quantity of food intake.

Notably, while physiological sensations associated with hunger, such as stomach pain, uneasy feeling or fatigue, were referenced in the definitions provided by ten studies^(7,24,25,28,29,32–35,42)^, none of the scales directly measured these experiences. These omissions reflect a lack of regard for children’s subjective experiences of hunger as the physiological, cognitive and emotional aspects of hunger have been reported to be more salient to children themselves^(93)^. Including child-reported physical and emotional indicators could improve both sensitivity and relevance of child hunger assessments. These methodological gaps in operationalisation and assessment of child hunger across studies underscore the need for more comprehensive and standardised approaches that account for the multidimensional nature of child hunger, including quantity, quality, severity, and physiological and emotional impacts of hunger.

The most common approach to measure child hunger was the use of a single question linking the *experience* of hunger to insufficient *quantities* of food in the home. While this approach was frequently used with child respondents for feasibility of administration and minimal participant burden, it has been critiqued for oversimplifying the operationalisation and measurement of hunger^(91)^. As discussed above, hunger as a complex construct with a range of indicators uniquely associated with social, physiological and structural dimensions of hunger is not likely captured through the use of single-item measures.

Another key inconsistency was the temporality of hunger across definitions and measurement approaches in the included studies. While some definitions and measures referred to episodic or short-term hunger^(26)^, most focused on chronic or long-term experiences of hunger among children^(27,32,39)^. Similarly, the measures employed a range of reference periods, from short-term intervals (e.g. past weekend, past 7 d or past 30 d)^(23,27,28,34,69,71,72,74–76,85–89)^ to longer-term periods (e.g. the past 12 months)^(26,29,32,35,50,52–56,58,61,68,70,90)^. Despite its implications for practice, the importance of clearly defining the temporality of child hunger is often overlooked in the literature. For example, a child who is hungry only during school holidays *v*. one who is persistently underfed all year would both count as ‘food-insecure’ using a 12-month measure, despite their need for different interventions. To address this, future research should desegregate hunger experiences by duration and frequency, distinguishing between acute, intermittent and chronic hunger. Additionally, studies should assess contextual factors contributing to fluctuations in food access and resulting child hunger (e.g. school closures, seasonal income changes or household shocks) to better target interventions based on the timing and persistence of child hunger.

The level of measurement was rarely conceptualised in the definitions of child hunger, with only three studies explicitly defining it as an individual-level construct^(24,25,32)^. However, the administration of child hunger scales varied across studies. Those aiming to assess hunger at the individual child level typically included children as respondents^(6,29,32,35,41,49–57,60,61,63–68)^, whereas studies examining both food insecurity and hunger at the household level predominantly relied on caregiver-reported measures^(25,66,69–72)^. This finding is consistent with a recent scoping review on food insecurity, which reported that the measurement of child food insecurity has ‘predominantly [been] reported by adult respondents on behalf of children’^(91)^. This raises critical questions about whose perspectives are being captured in the assessment of child hunger and food insecurity. Research increasingly shows that children may have distinct experiences of food insecurity that may not be fully known or reported by adults in the household^(91,93)^. Children, for example, have been found to be acutely aware of household food shortages and may internalise or hide their experiences of hunger and food insecurity to protect their caregivers^(73,93,94)^. As such, relying solely on caregiver reports may obscure children’s lived realities^(93)^ and underestimate the true prevalence of hunger and food insecurity among children^(91)^. To address this gap, future research should prioritise the inclusion of both child-and caregiver-reported measures. Using parallel instruments at both the individual and household levels can offer a more comprehensive and reliable understanding of child hunger and food insecurity^(91)^.

Beyond measurement challenges, geographic gaps were also observed. Most of the included studies were conducted in the USA, Canada, Malaysia and South Africa. While child hunger is a global challenge, the countries with the highest prevalence of food insecurity in 2023 were Somalia, Afghanistan, Syrian Arab Republic, Haiti, Yemen, Sierra Leone, Nigeria, Liberia, Mauritania, Central African Republic, Lesotho and Vanuatu^(95)^. Countries with the highest prevalence of childhood obesity, on the other hand, include the Cook Islands, Wallis and Futuna, Niue, Tonga, and Samoa, among other countries of Polynesia and Micronesia^(96)^. However, our review did not find any relevant studies conducted in these countries. Future research should be prioritised in countries with high levels of food insecurity and child hunger.

The current review is not without limitations. Only English language studies were included, which could have resulted in the exclusion of potentially relevant studies. Additionally, the inclusion of only a small number of NGO reports (*n* 3) may reflect the specificity of our narrowly focused inclusion criteria. Lastly, this rapid review used single-reviewer screening and data extraction, with a second reviewer checking a subset of studies, and did not include a formal quality assessment. These choices may limit the comprehensiveness of the study selection process and preclude conclusions regarding the quality of included studies; however, they are unlikely to introduce systematic bias because the review focuses on identifying and describing the range of methods used to measure child hunger, rather than estimating effects or evaluating the comparative performance of different approaches. Furthermore, the use of a pre-registered protocol, systematic searches across multiple databases and standardised study selection process support the validity of the findings. Results should be interpreted in light of these considerations.

### Conclusion

This rapid review sought to understand what is meant by ‘child hunger’ and how it is measured through a rapid and systematic investigation of research. Despite recommendations from CNSTAT to clearly define and operationalise child hunger and to differentiate between child hunger and household food insecurity, these principles are inconsistently applied in extant child hunger research. Our review highlights persistent and significant variations in how child hunger is conceptualised and measured across studies, with differences in definitions, reference periods, levels of reporting and the inclusion of key dimensions such as quantity, quality, severity and temporality. Moreover, the dominance of caregiver-reported measures raises concerns about the extent to which children’s unique experiences are accurately captured. To advance research and policy, future work should prioritise child-centred, culturally relevant tools that reflect both the multiple dimensions and lived experience of child hunger.

## Supporting information

Mooney et al. supplementary material Mooney et al. supplementary material

## Supplementary material

For supplementary material accompanying this paper visit https://doi.org/10.1017/S1368980026102195
