# Supplementary material for: Conceptualisation and measurement of child hunger: a rapid review
Source: Public Health Nutr. 2026 Mar 25;29(1):e71. doi: 10.1017/S1368980026102195 (PMC13087978; doi:10.1017/S1368980026102195)
Supplement: Mooney et al. supplementary material [file S1368980026102195sup001.docx]

**Supplementary Material**

Annexure 1: Example search strategy for Web of Science

Annexure 2: Supplementary Table 1. Constructs measured by the child hunger measures

## Annexure 1

### Example search strategy for Web of Science

*TOPIC field only, limit to English, 2000-*

(adolescen* OR baby OR babies OR boy* OR child* OR girl* OR infant* OR juvenile* OR learner* OR minor* OR pediatric* OR paediatric* OR pupil* OR schoolboy* OR schoolgirl* OR student* OR teen* OR young* OR youth*) NEAR/3 (hunger OR hungry).

## Annexure 2

### Supplementary Table 1. Constructs measured by the child hunger measures

| **Measures used to assess child hunger** | **Frequency of hunger due to food insecurity** | **Inadequate or reduced food intake due to limited resources** | **Food insecurity** | **Low quality of food due to limited resources** | **Severity of hunger** | **Frequency of hunger** | **Stunting** | **Responsiveness to hunger** | **Hidden Hunger** | **Number of indicators assessed in a single measure** |
| --- | --- | --- | --- | --- | --- | --- | --- | --- | --- | --- |
| Radimer/Cornell Hunger and Food Insecurity Instrument |  |  |  |  |  |  |  |  |  | 4 |
| Question - ‘During the past 30 days, how often did you go hungry because there was not enough food in your home?’ |  |  |  |  |  |  |  |  |  | 1 |
| US Household Food Security Survey Module (HFSSM) |  |  |  |  |  |  |  |  |  | 3 |
| Community Childhood Hunger Identification Project (CCHIP) index |  |  |  |  |  |  |  |  |  | 3 |
| Variations of question – ‘Has this child ever experienced being hungry because the family has run out of food or money to buy food?’ |  |  |  |  |  |  |  |  |  | 1 |
| Other* |  |  |  |  |  |  |  |  |  | 4 |
| (Looking back at your life), was there a period when you suffered from hunger? / how frequently children in the household went hungry over a 30-day period. |  |  |  |  |  |  |  |  |  | 1 |
| Question – ‘Some young people go to school or to bed hungry because there is not enough food at home. How often does this happen to you?’ |  |  |  |  |  |  |  |  |  | 1 |
| Question – How often (every day, almost every day, sometimes, never) you feel hungry when you arrive at school. |  |  |  |  |  |  |  |  |  | 1 |
| Question - When you were a child before age 17 was there ever a time when your family did not have enough food to eat? |  |  |  |  |  |  |  |  |  | 1 |
| Picture measures |  |  |  |  |  |  |  |  |  | 4 |
| Household Food Insecurity Access Scale (HFIAS) |  |  |  |  |  |  |  |  |  | 3 |
| Household Hunger Scale (HHS) |  |  |  |  |  |  |  |  |  | 1 |
| Modified/adapted version of Radimer/Cornell Hunger and Food Insecurity Instrument for an interview |  |  |  |  |  |  |  |  |  | 1 |
| Kindergarten Readiness Inventory (KRI) |  |  |  |  |  |  |  |  |  | 1 |
| Food Power Scale |  |  |  |  |  |  |  |  |  | 1 |
| Food Security battery of questions |  |  |  |  |  |  |  |  |  | 2 |
| Food and Nutrition Technical Assistance (FANTA) Project's Food Insecurity Access Scale (FIAS) |  |  |  |  |  |  |  |  |  | 3 |
| **Number of scales assessing different constructs** | 10 | 7 | 6 | 5 | 3 | 2 | 1 | 1 | 1 |  |
